# Efficacy of *Ageratum conyzoides* extracts against *Giardia duodenalis* trophozoites: an experimental study

**DOI:** 10.1186/s12906-020-2860-6

**Published:** 2020-02-28

**Authors:** Ai-rada Pintong, Jiraporn Ruangsittichai, Sumate Ampawong, Kanthinich Thima, Patchara Sriwichai, Narumon Komalamisra, Supaluk Popruk

**Affiliations:** 1 Department of Medical Entomology, Faculty of Tropical Medicine, Mahidol University, Ratchawithi Road, Ratchathewi, Bangkok, 10400 Thailand; 2 Department of Tropical Pathology, Faculty of Tropical Medicine, Mahidol University, Ratchawithi Road, Ratchathewi, Bangkok, 10400 Thailand; 3 Department of Protozoology, Faculty of Tropical Medicine, Mahidol University, Ratchawithi Road, Ratchathewi, Bangkok, 10400 Thailand

**Keywords:** *Giardia duodenalis*, *Ageratum conyzoides*, Crude extract, Organelles

## Abstract

**Background:**

*Giardia duodenalis* causes giardiasis in humans, particularly in developing countries. Despite the availability of treatments, resistance to some of the commercial anti-*Giardia* drugs has been reported in addition to their harmful side effects. Therefore, novel treatments for giardiasis are required. In this study, we aimed to assess the in vitro activity of crude extracts of *Ageratum conyzoides* against *G. duodenalis* trophozoites.

**Methods:**

Plants were classified into three groups based on their flower colors: white (W), purple (P), and white–purple (W–P). Plants were separately cut into leaf (L) and flower (F) parts. Changes in internal organelle morphology of trophozoites following exposure to crude extracts were assessed using transmission electron microscopy (TEM). In subsequent experiments, efficacy of the most active essential oils from crude extracts [half maximal inhibitory concentrations (IC_50_) ≤ 100 μg/mL] against *G. duodenalis* trophozoites was tested. In vitro anti-*Giardia* assays using essential oils were performed in the same way as those performed using crude extracts.

**Results:**

LW–P and FP extracts showed high activity (IC_50_ ≤ 100 μg/mL) against *G. duodenalis* trophozoites, with IC_50_ ± SD values of 45.67 ± 0.51 and 96.00 ± 0.46 μg/mL, respectively. In subsequent experiments, IC_50_ ± SD values of LW–P and FP essential oils were 35.00 ± 0.50 and 89.33 ± 0.41 μg/mL, respectively. TEM revealed the degeneration of flagella and ventral discs of *G. duodenalis* trophozoites following exposure to crude extracts.

**Conclusion:**

Crude LW–P and FP extracts of *A. conyzoides* showed the highest activity against *G. duodenalis*. Exposure to crude extract induced changes in the flagella and ventral discs of *G. duodenalis* trophozoites, which play important roles in attachment to the surface of mucosal cells. Our results suggest that the tested extracts warrant further research in terms of their efficacy and safety as giardiasis treatment.

## Background

*Giardia duodenalis* (syn. *G. lamblia* or *G. intestinalis*) is a common enteric protozoan that causes giardiasis in humans and animals. The greatest burden of giardiasis worldwide is found in developing countries, where poor sanitary conditions and ineffective water treatment are common [1]. Nearly 280 million people worldwide are infected annually [2–5]. In Thailand, the prevalence of giardiasis in humans has been reported to be 0.4–37.7% in different populations and locations [6–20]. Giardiasis leads to mortality and morbidity in the elderly, travelers, and patients with immune system defects [21, 22]. Giardiasis symptoms vary from asymptomatic cases to chronic diarrhea [23]. In cases of severe infection in children, it may lead to malnutrition and affect mental and physical development [24]. Giardiasis in patients with HIV/AIDS can lead to acute or chronic diarrhea [25–28].

Partial failure in giardiasis treatment due to drug resistance has been reported to occur in endemic areas [1]. Metronidazole is the first-line treatment for giardiasis, although adverse side effects have been reported; metallic taste, headache, dry mouth and, to a lesser extent, nausea, glossitis, urticaria, pruritus, and dark colored urine [29, 30]. Moreover, carcinogenic, teratogenic, and embryogenic properties of metronidazole have been reported [31, 32]. Therefore, search for novel agents to treat giardiasis has accelerated.

Plant products, such as crude extracts and essential oils, are potential alternative agents being explored for the development of novel antimicrobial drugs. One of their advantages is that they have low toxicity in mammalian cells [33]. Plants belonging to the family Compositae show medicinal properties and contain numerous compounds, some of which may have potential as novel drug sources [34, 35]. Moreover, crude extracts and compounds isolated from these plants have shown antiprotozoal activity [35]. Some Compositae species, including *Chromolaena odorata* and *Tithonia diversifolia*, have been reported to show antimalarial activities and have been used in traditional medicine [36–38].

*Ageratum conyzoides* is a common annual herbaceous weed belonging to the family Compositae and has a long history of traditional medicinal use [39]. It is a tropical plant commonly found in Central America, the Caribbean, Southeast Asia, South China, India, West Africa, Australia, and South America [40, 41]. In many countries, it is utilized in folk medicine, such as that for skin diseases, wound healing, diarrhea, and navel pain in Nigerian children [41–43]. Phytochemical investigations of this plant have revealed pharmacological and insecticidal properties conferred by a number of its secondary metabolites [41, 44]. Whole plant extracts have shown inhibitory action against bacteria and mosquitoes [45]. Aqueous and ethanolic extracts of *A. conyzoides* leaves were tested for their activity against the intestinal worm *Heligmosomoides bakeri*, with ethanolic extracts being particularly efficient against this worm [46]. However, no studies thus far have tested the properties of this plant against pathogenic intestinal protozoa, such as *G. duodenalis*.

Therefore, in this study, we aimed to investigate the antiprotozoal properties of *A. conyzoides* extracts. Efficacy of six types of crude extracts, including those from leaves of white (LW), purple (LP), or white–purple flowered (LW–P) plants and flowers of white (FW), purple (FP), and white–purple flowered (FW–P) plants as well as two types of essential oils from LW–P and FP plants, was tested against *G. duodenalis* trophozoite. Changes in internal organelle morphology of trophozoites following exposure to crude extracts were assessed using transmission electron microscopy (TEM). Our result demonstrated the efficacy and organelle targets of *A. conyzoides* extracts against *G. duodenalis* and provided basis for the development of novel therapeutic agents against giardiasis in the future.

## Methods

### Plant collection

We reviewed the characteristics of *A. conyzoides* such as flower, leaf, stem, and areas of distribution. In March 2016–2017, we visited wasteland in Chiang Khong district in Chiang Rai province (20°15′36′′ N 100°24′24′′ E) of Thailand, where this plant commonly grows. This plant is a type of weed that can grow anywhere. The wastelands were owned by residents of villages nearby. The owners readily agreed to plant collection because this is a weed. In the wild, flowers of three colors—white (W), purple (P), and white–purple (W–P)—were observed. They were identified plant species by Mrs. Parinyanoot Klinratana, a researcher at the Department of Botany, Faculty of Science, Chulalongkorn University, Thailand. The results represented that the flowers of three colors were the same plant. Voucher specimens are deposited in the Professor Kasin Suvatabhandhu Herbarium (Number 015854), Department of Botany, Faculty of Science, Chulalongkorn University, Thailand.

### Plant extractions

#### Crude extractions

**Fig. 1:**
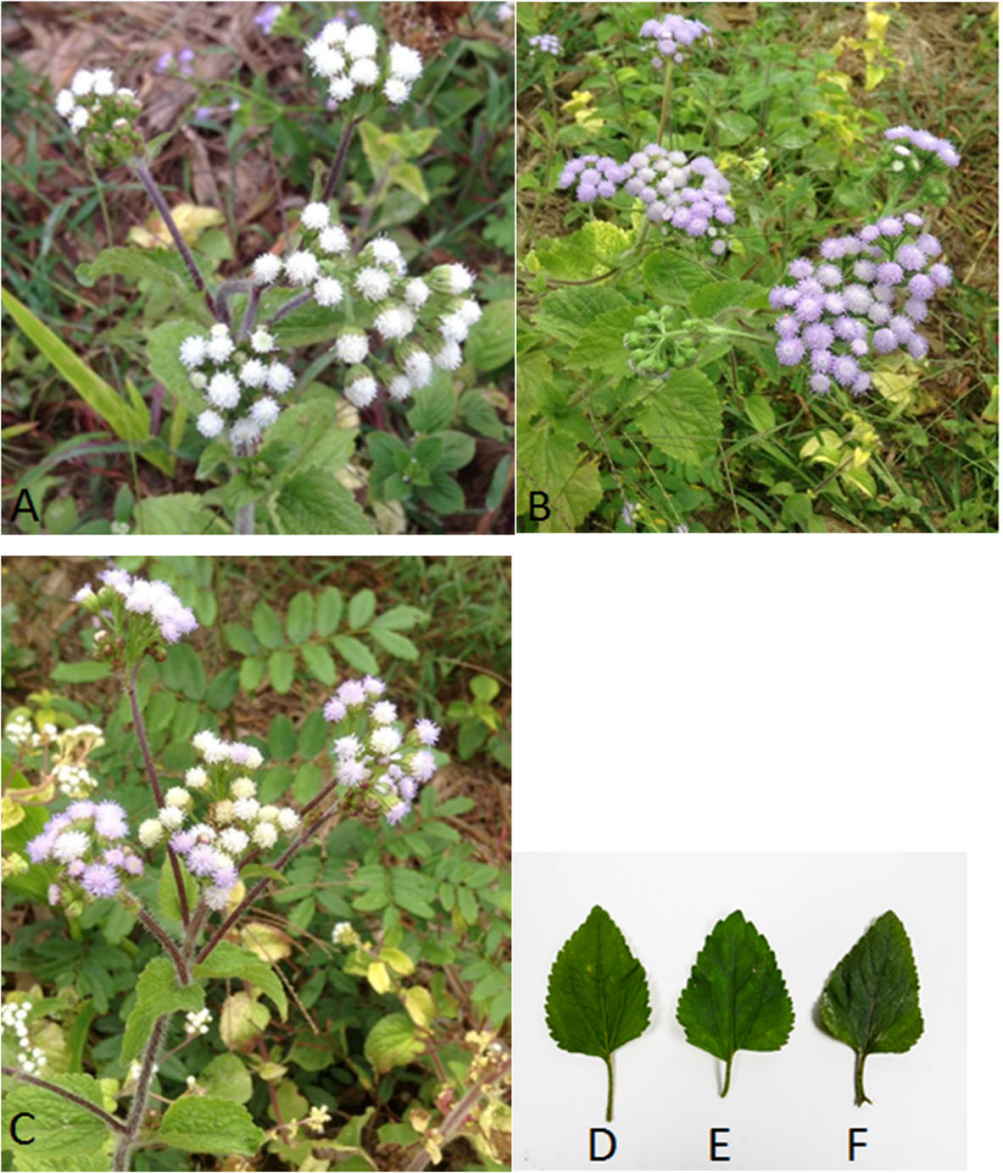
Different organs of *Ageratum conyzoides*: flowers of white flowered (**a**), flowers of purple flowered (**b**), flowers of white–purple flowered (**c**), leaves of white flowered (**d**), leaves of purple flowered (**e**), and leaves of white–purple flowered (**f**) plants

Fresh plants (LW, LP, LW–P, FW, FP, and FW–P) (Fig. 1) were dried at 60 °C in an oven for 7 days. The samples were stored in a cool and dry place. Dried plant material was pulverized and macerated using absolute ethanol as a solvent. The extract was filtered using Whatman filter paper (0.45 μm diameter), and solvent was removed using rotary vacuum evaporation (Heidolph, Germany). All dried crude extracts were stored at 4 °C until further use.

#### Essential oils

Fresh plants were suspended in distilled water and subjected to hydrodistillation for 3 h. Sodium sulfate (Na_2_SO_4_) was used to remove any trace of water from the essential oils, which were stored in dark glass vials at 4 °C until further use.

### *G. duodenalis* culture

Modified TYI-S-33 medium (Trypticase-yeast extract-iron-serum medium) was used for culturing *G. duodenalis* trophozoites modified by Keister’s modification [47]. Initially, *G. duodenalis* trophozoites were kept and maintained in the Department of Protozoology, Faculty of Tropical Medicine, Mahidol University. *G. duodenalis* trophozoites were grown in vitro under anaerobic conditions at 37 °C. After incubation for 24 h, cell growth and viability were examined every 2 days using inverse microscopy. Trophozoite-stage cultures were harvested once they reached the log phase (2–3 days), placed on ice for 10 min, and centrifuged (3500 rpm, at 4 °C for 7 min). Trophozoites were counted using a hemocytometer and used for subsequent experiments.

### In vitro anti-*Giardia* assay

Each crude extract was dissolved in 100% dimethyl sulfoxide (DMSO), and two-fold serial dilutions were made. Notably, 100% growth without extracts (non-treated) and 0.25% DMSO were used as negative control. Metronidazole (Sigma-Aldrich, St Louis, MO) without any extracts was used as positive control. Culture medium alone was used as a blank. For test groups, various concentrations of each crude extract were dissolved in 100% DMSO. Test samples along with negative and positive controls and the blank were added to 96-well microplates. Trophozoites at a density of 5 × 10^4^ were added to each well to make the final volume of 100 μL. The final concentration of DMSO was 0.25% in test groups (the final volume of 100 μL in 96-well microplates) and this concentration did not affect trophozoites (negative control). All experiments were performed in triplicates. The plates were sealed and incubated at 37 °C for 24 h under anaerobic conditions in 2.5-L Pack-Rectangular Jars (Mitsubishi Gas Chemical, Tokyo, Japan). After incubation for 24 h, 100 μL BacTiter-Glo™ Microbial Cell Viability Assay fluid was added to each well before trophozoite viability was recorded using luminescence. Percentage cell viability at each concentration of crude extract was determined using the following formula:

$$ \%\mathrm{cell}\ \mathrm{survival}=\left[\left(\mathrm{sample}\ \mathrm{luminescence}-\mathrm{culture}\ \mathrm{medium}\ \mathrm{luminescence}\right)/\left(\mathrm{non}-\mathrm{treated}\ \mathrm{control}\ \mathrm{luminescence}-\mathrm{culture}\ \mathrm{medium}\ \mathrm{luminescence}\right)\right]\times 100 $$

$$ \%\mathrm{inhibition}=100-\%\mathrm{trophozoites}\ \mathrm{that}\ \mathrm{survived} $$

The half maximal inhibitory concentration (IC_50_) was defined as the concentration of crude extract required to inhibit cell growth by 50%. The criteria used for defining the degree of activity of plant extracts in terms of inhibiting *Giardia* seemed to vary across test groups. Therefore, the following criteria proposed by Amaral et al. were used [48]: IC_50_ ≤ 100 μg/mL = highly active; 100 < IC_50_ ≤ 250 μg/mL = active, 250 < IC_50_ ≤ 500 μg/mL = moderately active; IC_50_ ≥ 500 μg/mL = inactive.

For subsequent experiments, the most active essential oils from crude extracts [IC_50_ ≤ 100 μg/mL (highly active)] were tested against *G. duodenalis* trophozoites. Essential oils from LW–P and FP plants were the most active. Next, in vitro anti-*Giardia* assays using essential oils were performed in the same way as those performed using crude extracts.

### Gas chromatography and mass spectroscopy (GC-MS) analysis

LW–P and FP essential oils were analyzed using an Agilent Technologies 6980 N GC chromatograph, equipped with a HP-5 MS capillary column (30 m × 0.25 mm × 0.25 μm) and interfaced to a mass spectrometer (5973 N). Helium was used as the carrier gas in the GC system, and the column temperature was increased by 7 °C/min between 100 °C and 300 °C. Samples were injected using the split mode, and the total run time was 46 min. MS conditions were measured at 70 eV at a mass range of m/z 50–600 amu. Components were identified based on peaks in gas chromatographic analyses and comparison of mass spectra with computer searches using Wiley 10th edition/NIST 2014 Combined Library.

### Ultrastructure analysis

TEM was performed to determine the ultrastructure of *G. duodenalis* trophozoites following exposure to crude extracts. Trophozoites treated with crude extracts leading to 50% cell death, 100% cell growth (negative control group), and 0% cell growth (positive control group) were fixed in 2.5% glutaraldehyde and 1% osmium tetroxide, dehydrated in graded ethanol, infiltrated in graded resin (LR white; EMS, USA), embedded in pure resin, and polymerized at 65 °C in an oven for 48 h. The specimens were cut into 100-nm-thick slices and stained with lead citrate and uranyl acetate. Using TEM (model HT7700, Hitachi, Japan), changes in ultrastructure were examined in at least 10 fields (300 *Giardia*/group), focusing on the nuclei, flagella, ventral discs, peripheral vesicles, chromatin, and shape, and percentage of abnormal cells per field was calculated (× 0.7 K magnification).

### Statistical analysis

Descriptive analysis (percentages) was used to describe sample data in this study. The mean IC_50_ ± standard deviations (SD) values for the crude extracts, essential oils and metronidazole were determined using SPSS version 18.0 (IBM, Armonk, NY).

## Results

Yields of six types of crude extracts were 17.41, 11.44, and 12.15% from LW, LP, and LW-P, respectively, and 10.30, 12.24, and 10.95% from FW, FP, and FW-P, respectively. After 24 h of treatment, IC_50_ ± SD values were 130.00 ± 0.30 (active), 463.08 ± 0.87 (moderately active), and 45.67 ± 0.51 (highly active) μg/mL from LW, LP, and LW–P, respectively, and 166.00 ± 0.45 (active), 96.00 ± 0.46 (highly active), and 207.00 ± 0.50 (active) μg/mL from FW, FP, and FW–P, respectively, and the values were dose dependent. Essential oil yields from LW–P and FP were low at 0.19 and 0.16%, respectively. IC_50_ ± SD values of the LW–P and FP essential oils were 35.00 ± 0.50 (highly active) and 89.33 ± 0.41 (highly active) μg/mL, respectively.

Chemical constituents of LW–P and FP essential oils were 32 and 35, respectively. Primary chemical components were chromene groups (precocene I, precocene II, and 6-vinyl-7-methoxy-2,2-dimethylchromene), followed by sesquiterpenes (β-caryophyllene, α-caryophyllene, germacrene D, copaene, caryophyllene oxide, and β-bourbonene) and monoterpenes (α-pinene, camphene, β-pinene, limonene, and endo-bornyl acetate) (Table 1). These three chemicals represented 80% of the components found in each essential oil.

**Table 1:** The chemical constituents and components of LW-P and FP essential oils of *A. conyzoides*, expressed as percent of total area

No.	Components	Essential oils groups	Plant types (number of constituents)	
			LW-P (32)	FP (35)
1	Precocene I	Chromene	48.04	42.86
2	β-caryophyllene	sesquiterpenes	20.60	21.49
3	Precocene II	Chromene	12.81	14.45
4	α-caryophyllene	sesquiterpenes	2.51	4.09
5	Germacrene D	sesquiterpenes	2.87	3.46
6	Copaene	sesquiterpenes	0.21	0.08
7	Caryophyllene oxide	sesquiterpenes	0.58	0.43
8	6-vinyl-7-methoxy-2,2-dimethylchromene	Chromene	0.17	0.17
9	α-pinene	monoterpenes		0.06
10	Camphene	monoterpenes		0.93
11	β-pinene	monoterpenes		0.05
12	Limonene	monoterpenes		0.14
13	β-bourbonene	sesquiterpenes	0.14	
14	endo-bornyl acetate	monoterpenes	0.17	

**Fig. 2:**
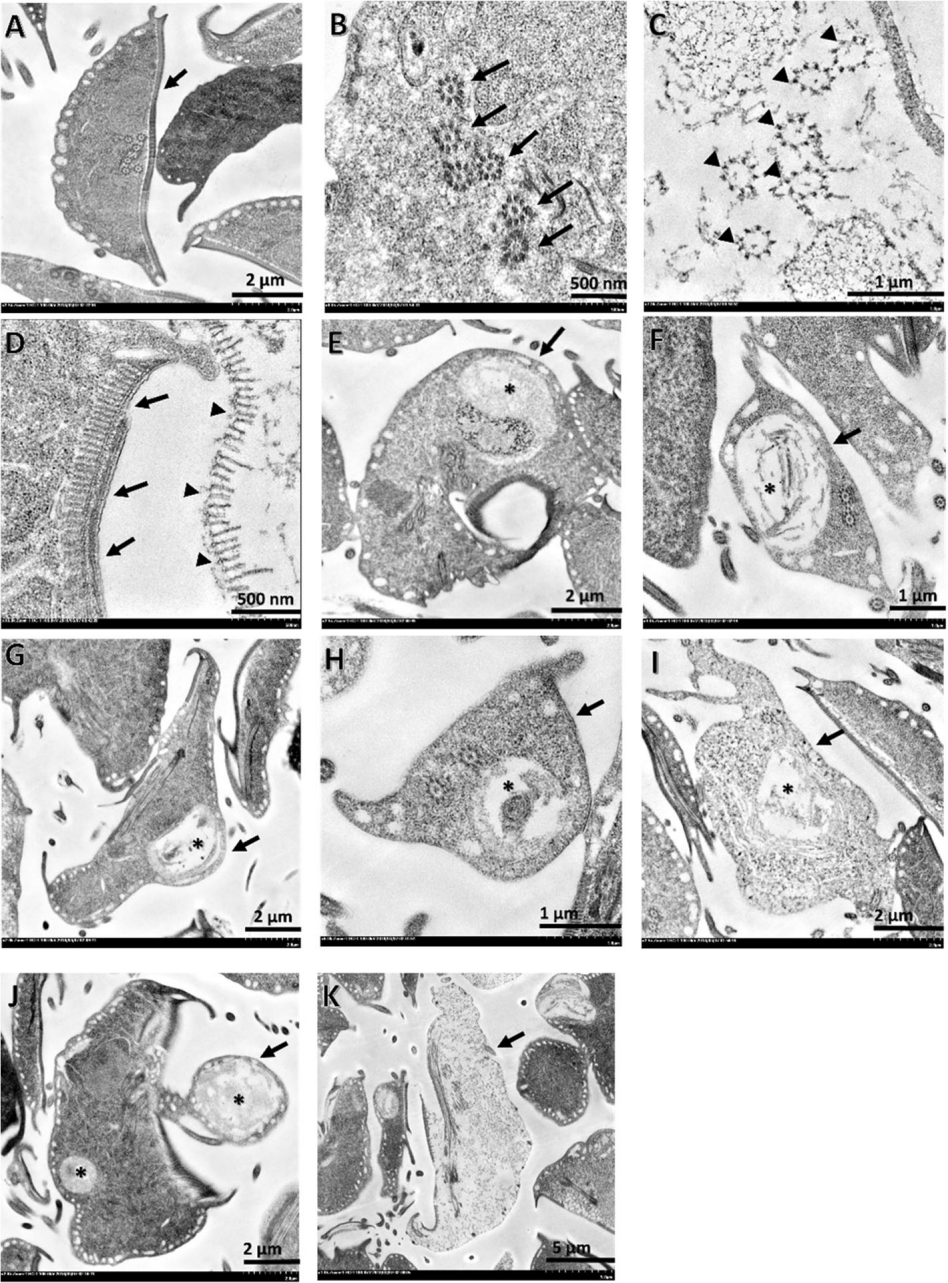
Morphological changes in ultrastructure of *Giardia* exposed to crude extracts of *Ageratum conyzoides* (LW–P and FP): normal architecture of *Giardia* (**a**; arrow) and its flagella (**b**; arrow) compared with degenerated flagella and ventral discs (**d**: normal; arrow and degenerated: arrowhead); nucleolar changes (**e**; star); vacuolation (**f**-**i**; star); unusual shape (**j**; arrow); and chromatin loss (**k**; arrow) were observed

*Giardia* trophozoites exposed to crude extracts, including LW–P and FP, showed ultrastructural changes compared with normal architecture when examined using TEM (Fig. 2a and b; arrow),

## Discussion

Several methods have been used to evaluate the cytotoxicity and viability of cells, including dye exclusion (trypan blue), colorimetric assays (3-[4,5-dimethylthiazol-2-yl]-2,5 diphenyl tetrazolium bromide), fluorometric assays (alamarBlue assay and CFDA-AM assay), and luminometric assays (ATP assays) [49]. Previous studies have investigated plant extract activity against *Giardia* and used dye exclusion and colorimetric assays to count viable cells [33, 46, 50–58]. In this study, a luminometric assay was selected for the detection of viable *Giardia* trophozoites exposed to crude extracts and essential oils from *A. conyzoides* because it is more sensitive and less susceptible to artifacts than are other methods of testing viability [49]. Crude extracts of LW–P (45.67 μg/mL) and FP (96.00 μg/mL) exhibited the highest activity against *G. duodenalis*. Notably, ethanolic extracts of different plant parts and different colored flowers of *A. conyzoides* exhibited different levels of efficacy against *Giardia*. Rayan et al. [57] tested the activity of methanolic and aqueous *Terminalia ferdinandiana* fruit extracts against *G. duodenalis* and found the IC_50_ values of 704 (inactive) and 143 (active) μg/mL, respectively (lower efficacy than this study). Calzada et al. [52] tested in vitro activity of 26 plants against *Giardia* and found that *Dorstenia contrajerva*, *Senna villosa*, and *Ruta chalepensis* were showed high activity against *Giardia*, with IC_50_ values of < 38 μg/mL. Compared with IC_50_ values reported in previous studies, high-to-moderate activities of crude extracts and essential oils were observed in this study [33, 46, 50–58].

In this study, the IC_50_ values of essential oils were slightly lower than those of crude extracts perhaps because of purer active compounds in essential oils when extracted by hydrodistillation. Moreover, a leaf decoction of *A. conyzoides* has been used to treat patients with diarrhea in Bangladesh [59]. The crude extracts may be more practical to use than are essential oils, particularly in remote areas and areas with high prevalence of parasitic infections*.* The solvent selected for extraction is important to avoid contamination and to ensure the safety of treatment. In this study, we did not test the cytotoxicity of tested extracts. However, a previous study has reported that leaf extract of *A. conyzoides* using hydroalcohol (ethanol 90: water 10) was not harmful when administered orally to rats [60], and the solvent used in that study was somewhat similar to the one used in this study.

The primary chemical components of LW–P and FP essential oils were chromenes, followed by sesquiterpenes and monoterpenes. Notably, FP essential oil contained many monoterpenes, which may have reduced its activity compared with LW–P essential oils in this study. Assumedly, monoterpenes have more analgesic properties than other chemical groups. Machado et al. [61] have reported that essential oils rich in monoterpenes (carvacrol) from *Thymbra capitata* and *Origanum virens* showed the greatest efficiency against *G. duodenalis*. However, this result is not consistent with previous reports because of differences in the main chemical components across monoterpene group. Essential oils from plants have been shown to exhibit anti-helminthic, anti-tumor, anti-inflammatory, nematocidal, insecticidal, and anti-parasitic activities [62–67]. These functions may be related to the diverse chemical components found in essential oils [68]. Two mechanisms of action of essential oils can explain their anti-parasitic activities: direct anti-parasitic action and immunomodulatory properties [62]. However, details of mechanisms underlying these actions of essential oils remain unknown [69].

TEM revealed that the ultrastructure of *G. duodenalis* trophozoits treated with or without crude extracts showed different internal structures between the two groups. Structural alterations including those in flagella, ventral discs, nuclei, cellular vacuoles, shapes, and chromatin were observed (Fig. 2). Crude extracts may have caused cell death by reducing the attachment ability by degenerating the flagella and ventral discs (Fig. 2c, d), which represent similar targets as those of commercial anti-*Giardia* drugs [70, 71]. These structures play a significant role in the attachment of protozoans to the surface of intestinal cells [72]. Similarly, nuclei showed altered shapes (Fig. 2e). The ultrastructure of *G. duodenalis* treated with or without essential oils warrant further investigation to clarify the significance of morphological changes in organelles.

## Conclusions

LW–P and FP from *A. conyzoides* were more effective against *Giardia* than the other tested extracts. Thus, *A. conyzoides* may be a potential source of anti-*Giardia* drugs. Moreover, exposure to these extracts changed the ultrastructure of *Giardia trophozoites*, such as flagella and ventral discs, which are the structures targeted by commercial anti-*Giardia* drugs. Therefore, *A. conyzoides* extracts, particularly from LW–P and FP plants, warrant further investigation in terms of their efficacy and safety as giardiasis treatment.

## Supplementary information

**Additional file 1.**

## Data Availability

The datasets used and/or analyzed during the present study are available from the corresponding author on reasonable request.

## Abbreviations

DMSO: Dimethyl sulfoxide
FP: Flowers of purple flowered plants
FW: Flowers of white flowered plants
FW–P: Flowers of white–purple flowered plants
GC-MS: Gas chromatography-mass spectrometry
IC_50_: Half maximal inhibitory concentration
LP: Leaves of purple flowered plants
LW: Leaves of white flowered plants
LW–P: Leaves of white–purple flowered plants
TEM: Transmission electron microscopy
