# Supplementary material for: Efficacy of Ageratum conyzoides extracts against Giardia duodenalis trophozoites: an experimental study
Source: BMC Complement Med Ther. 2020 Feb 28;20:63. doi: 10.1186/s12906-020-2860-6 (PMC7076862; doi:10.1186/s12906-020-2860-6)
Supplement: Supplementary file 1 — Additional file 1. [file 12906_2020_2860_MOESM1_ESM.pptx]

## Slide 1
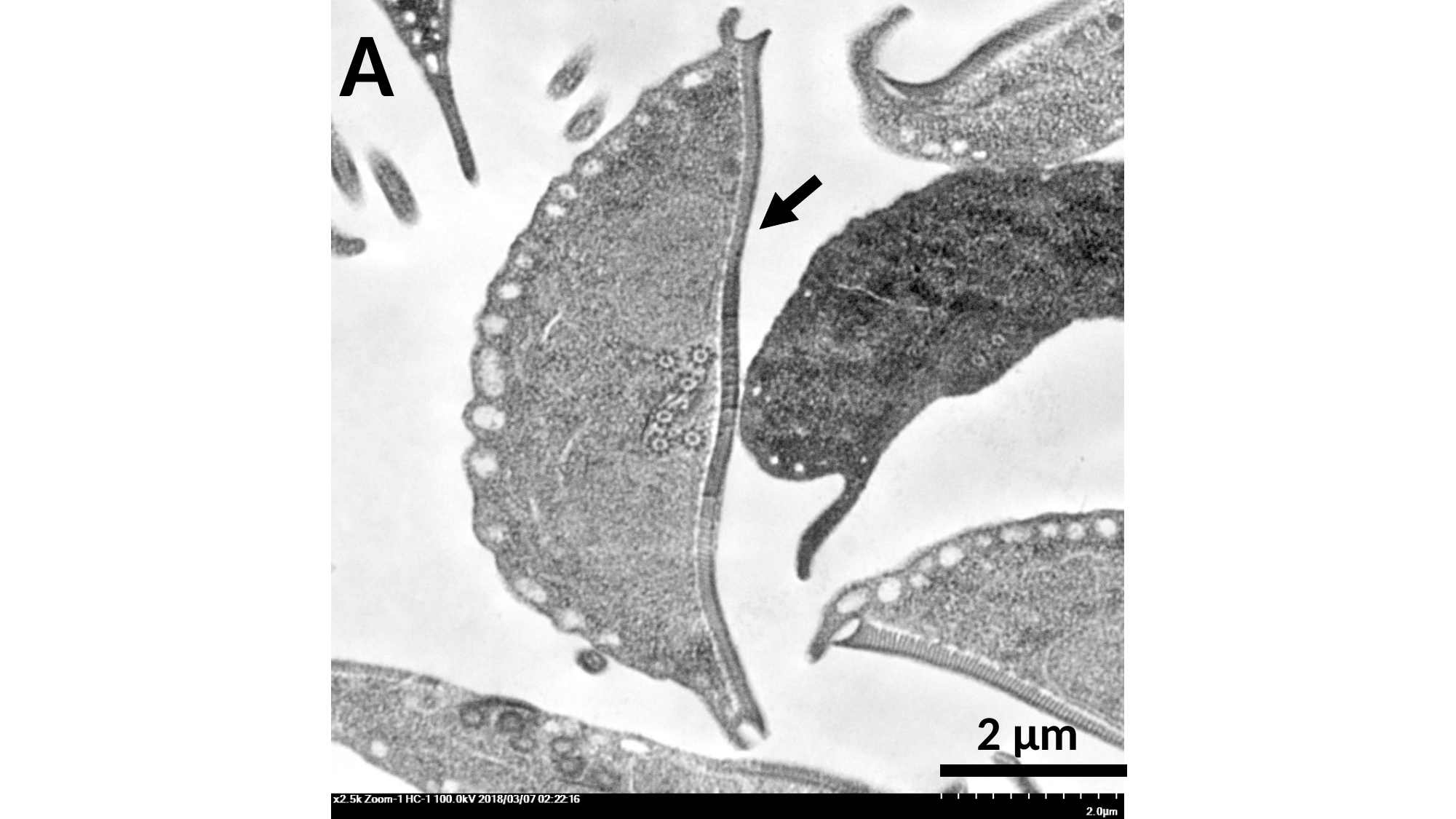

A
2 µm

## Slide 2
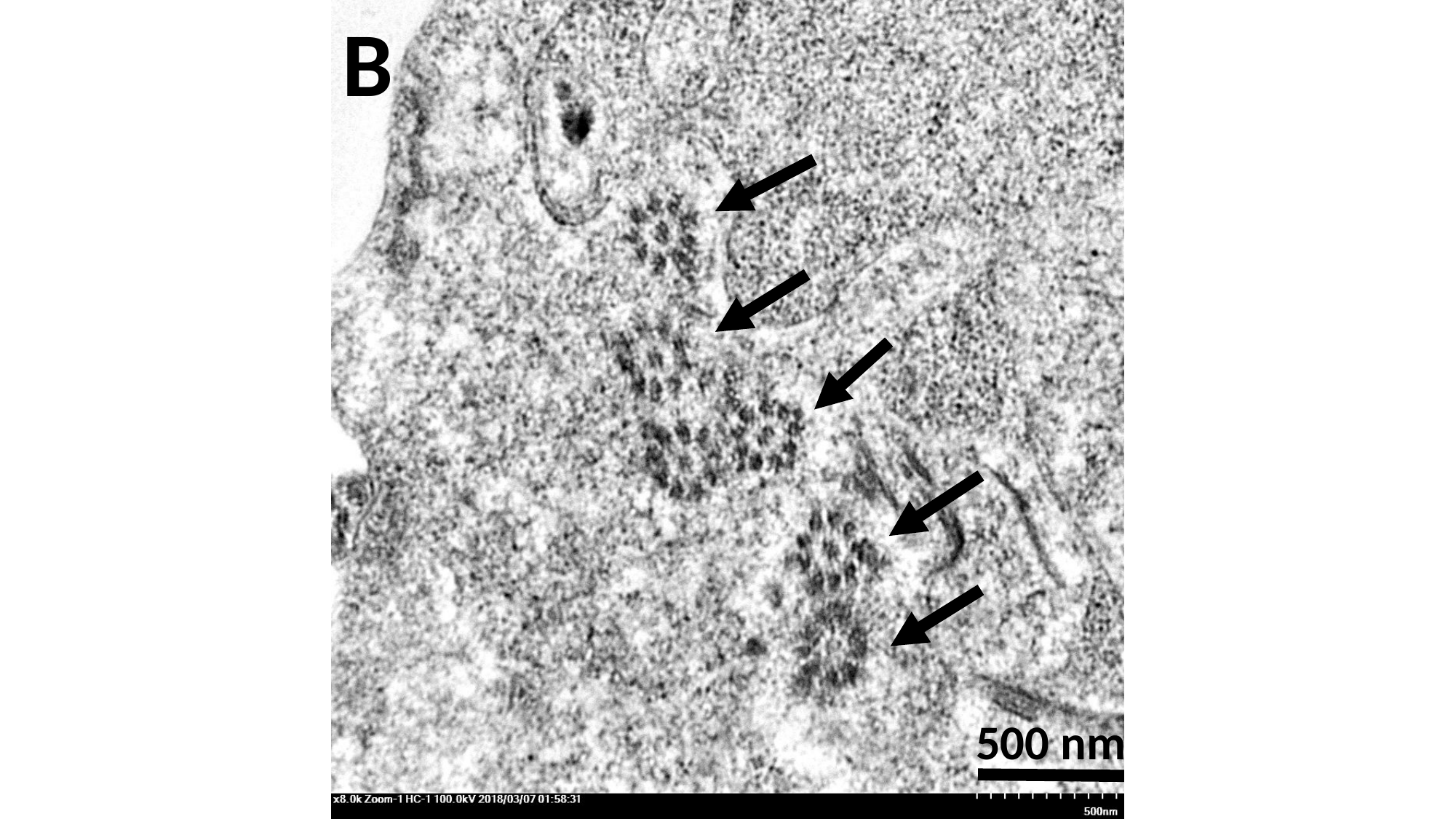

B
500 nm

## Slide 3
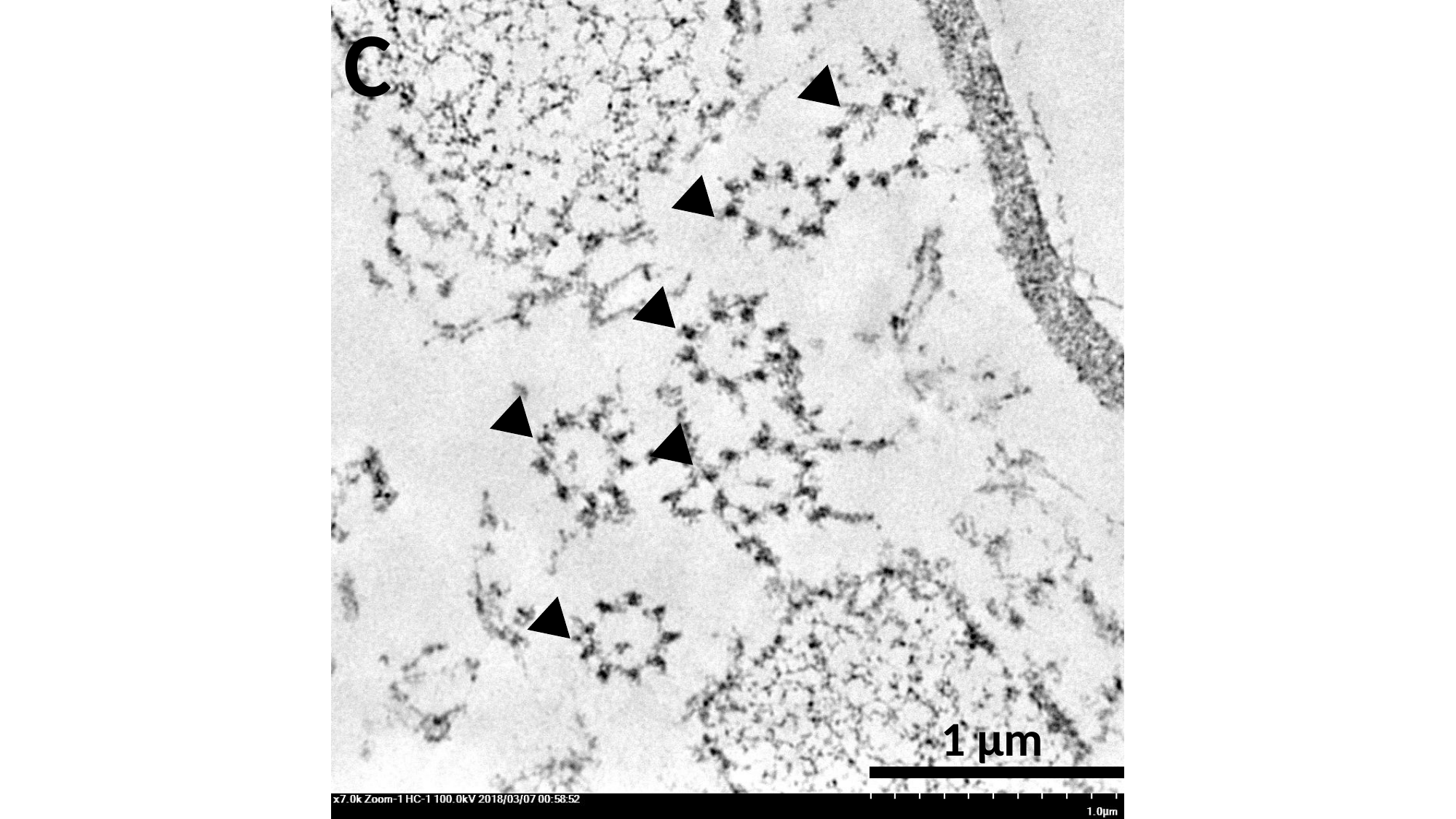

C
1 µm

## Slide 4
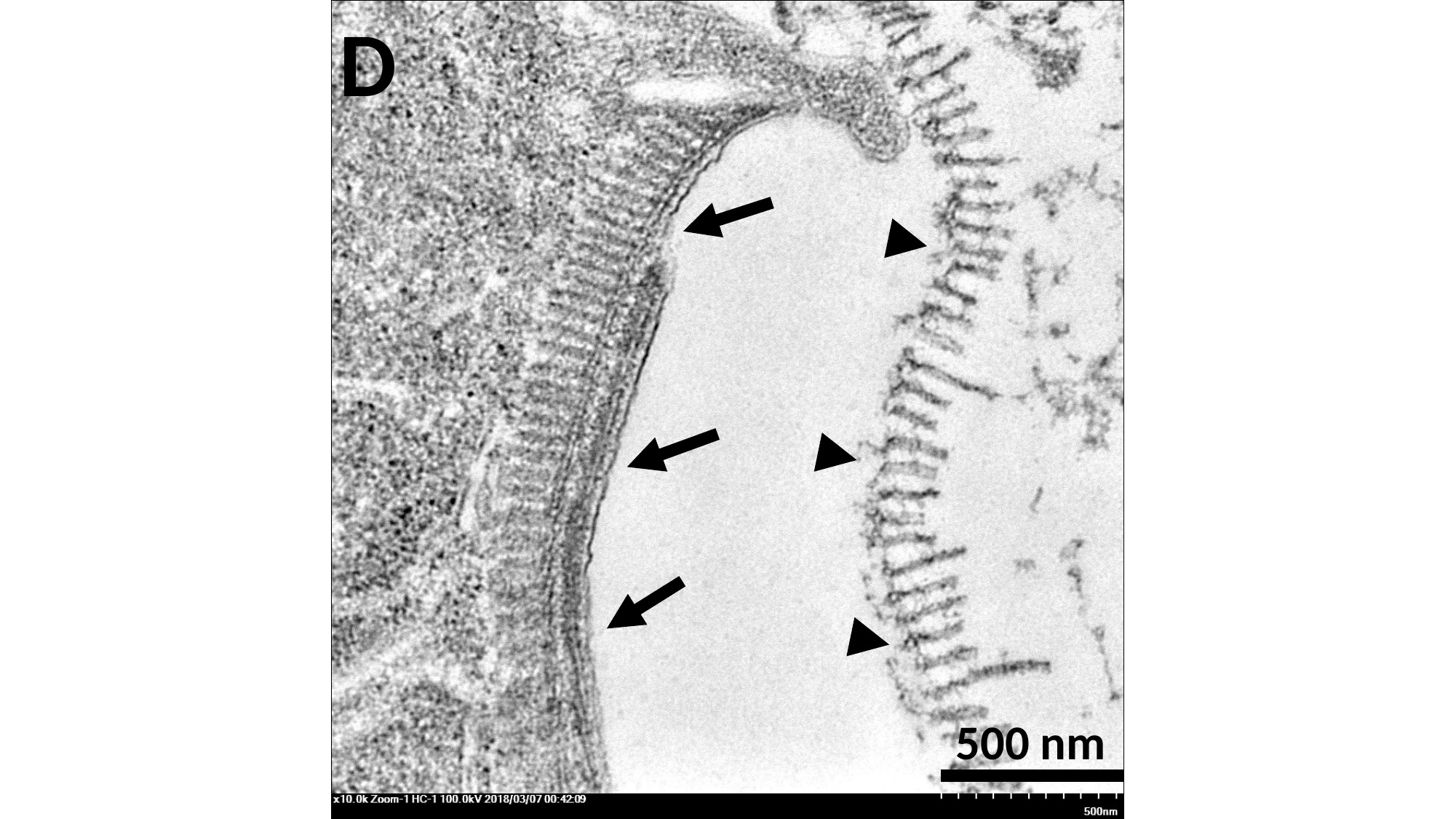

D
500 nm

## Slide 5
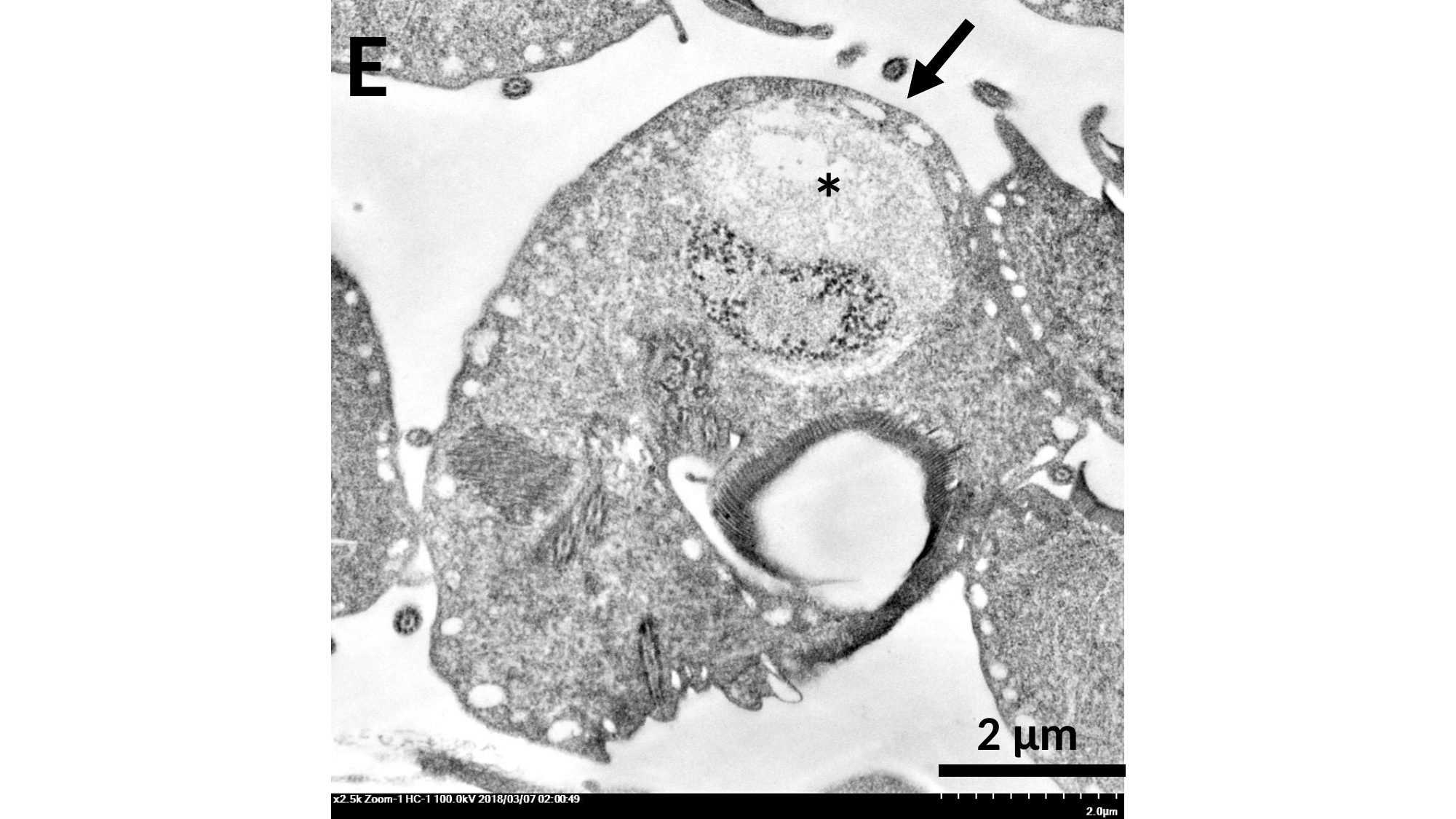

E
*
2 µm

## Slide 6
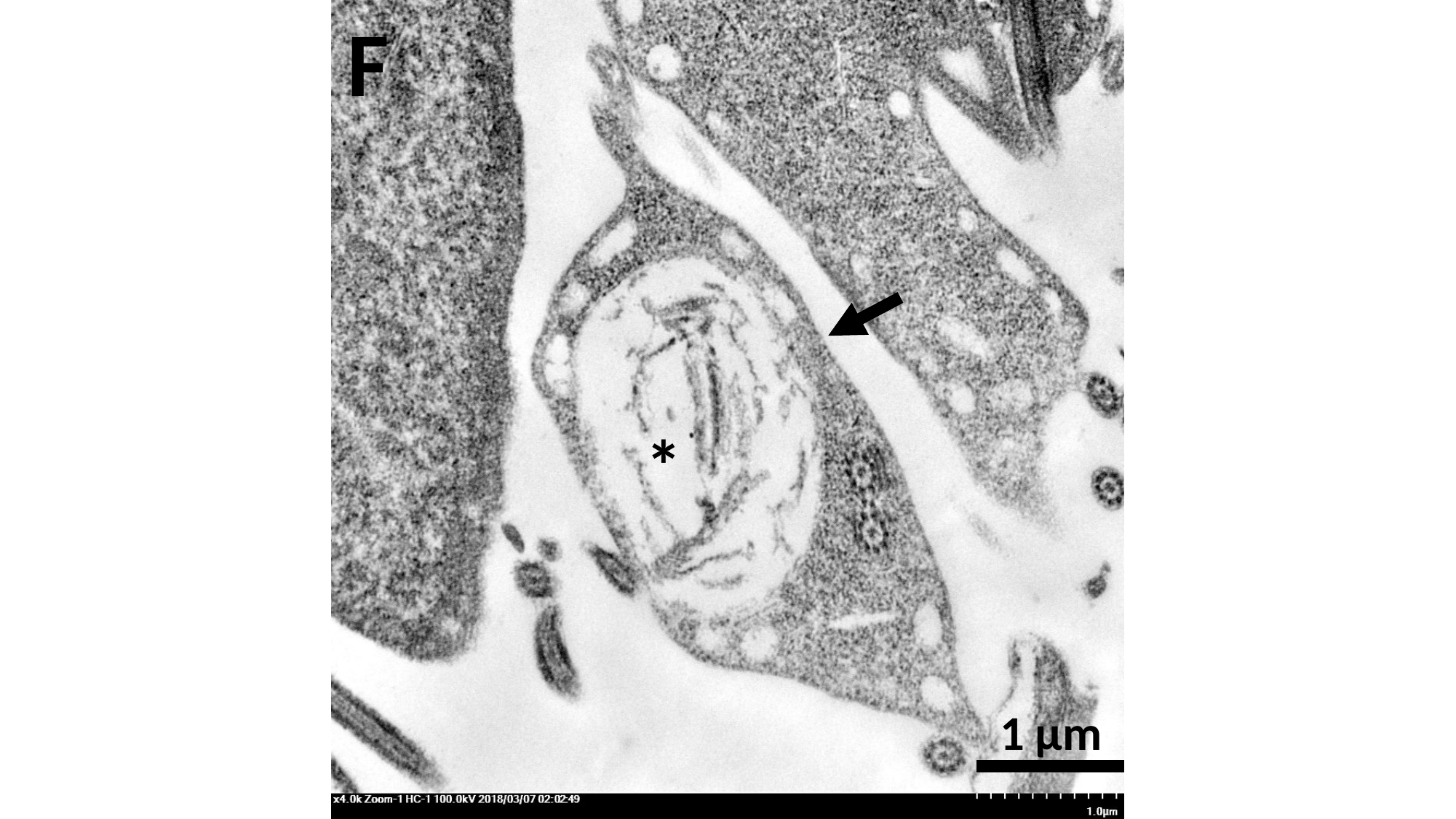

F
*
1 µm

## Slide 7
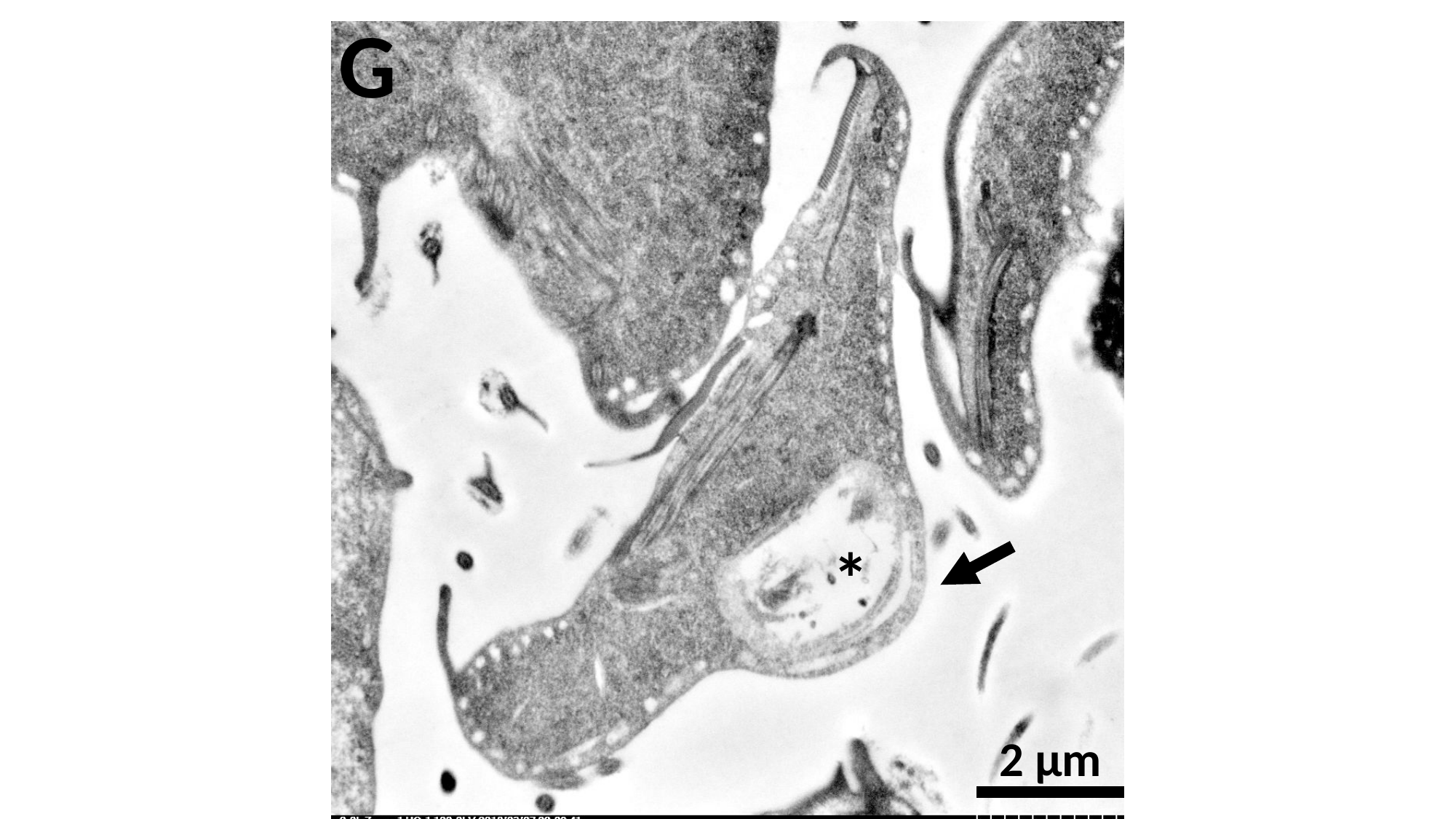

G
*
2 µm

## Slide 8
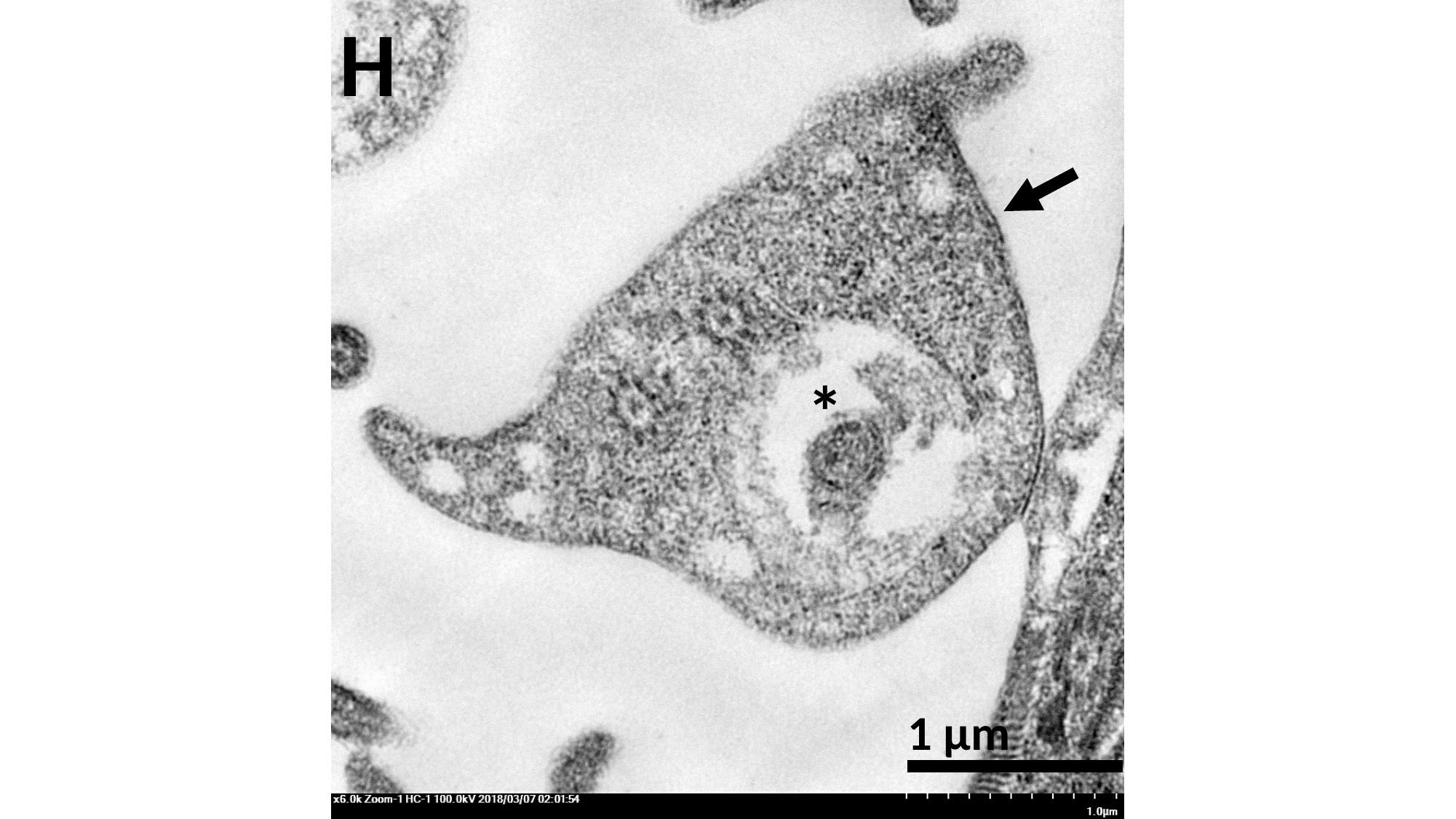

H
*
1 µm

## Slide 9
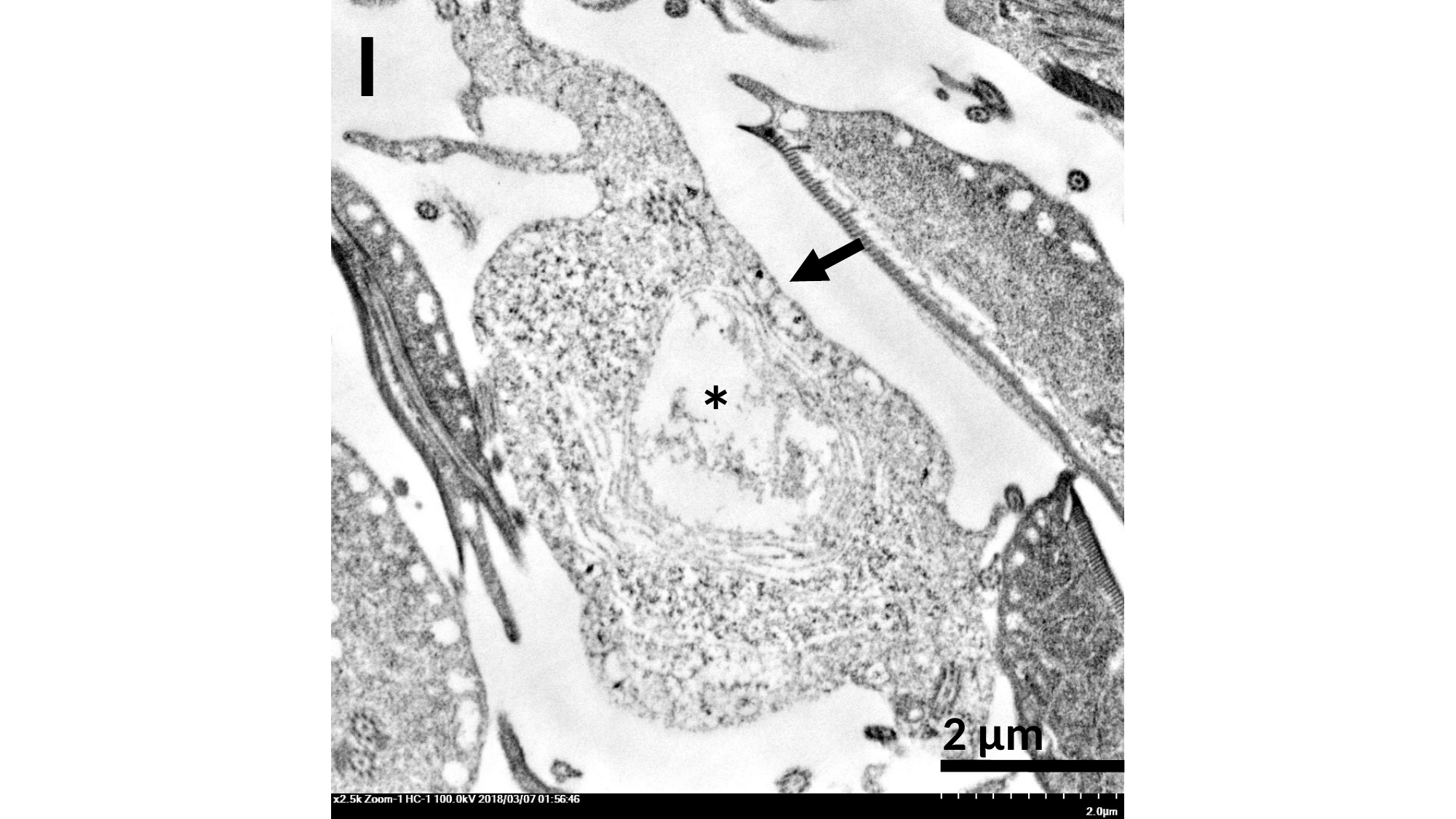

I
*
2 µm

## Slide 10
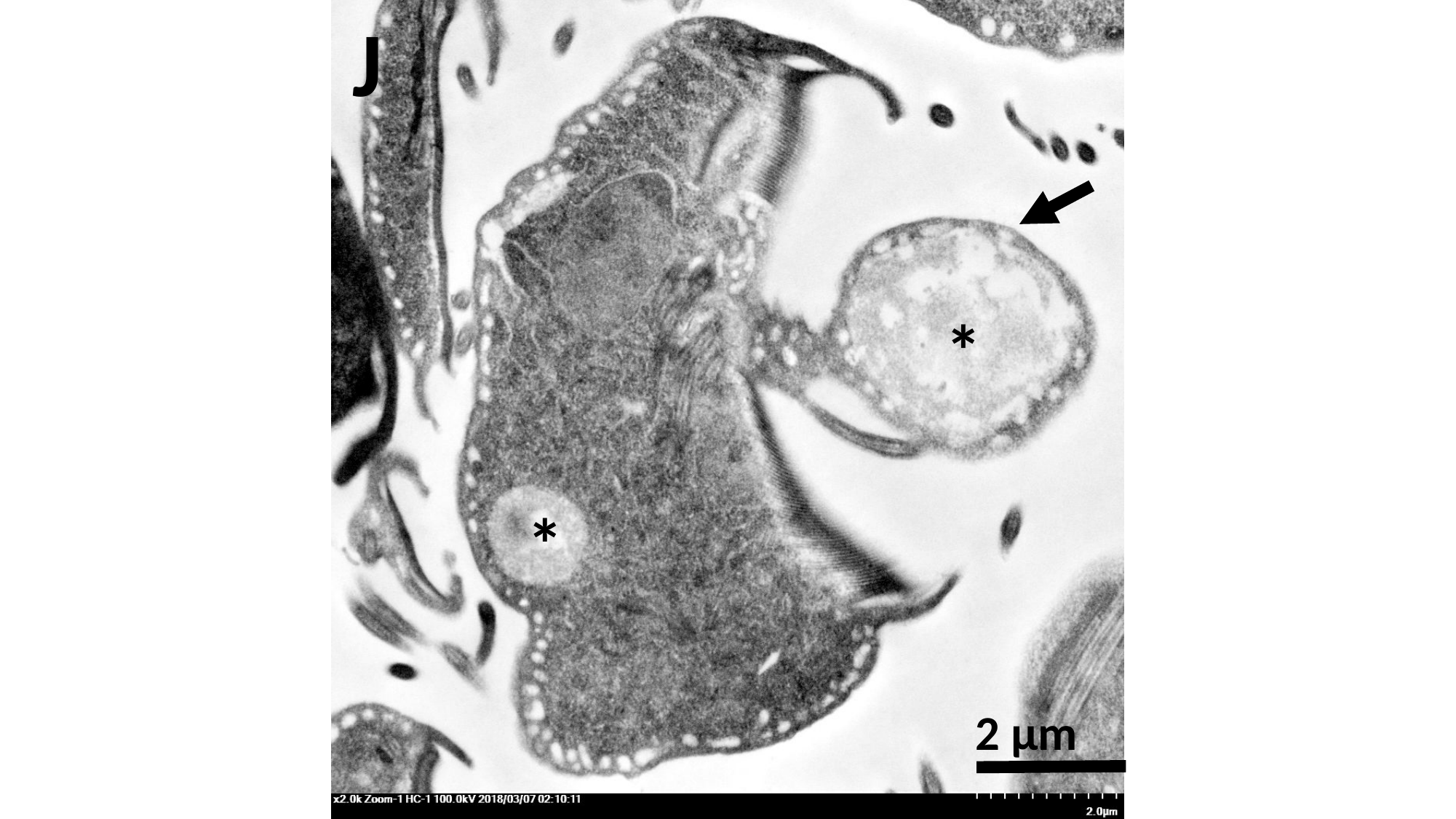

J
*
*
2 µm

## Slide 11
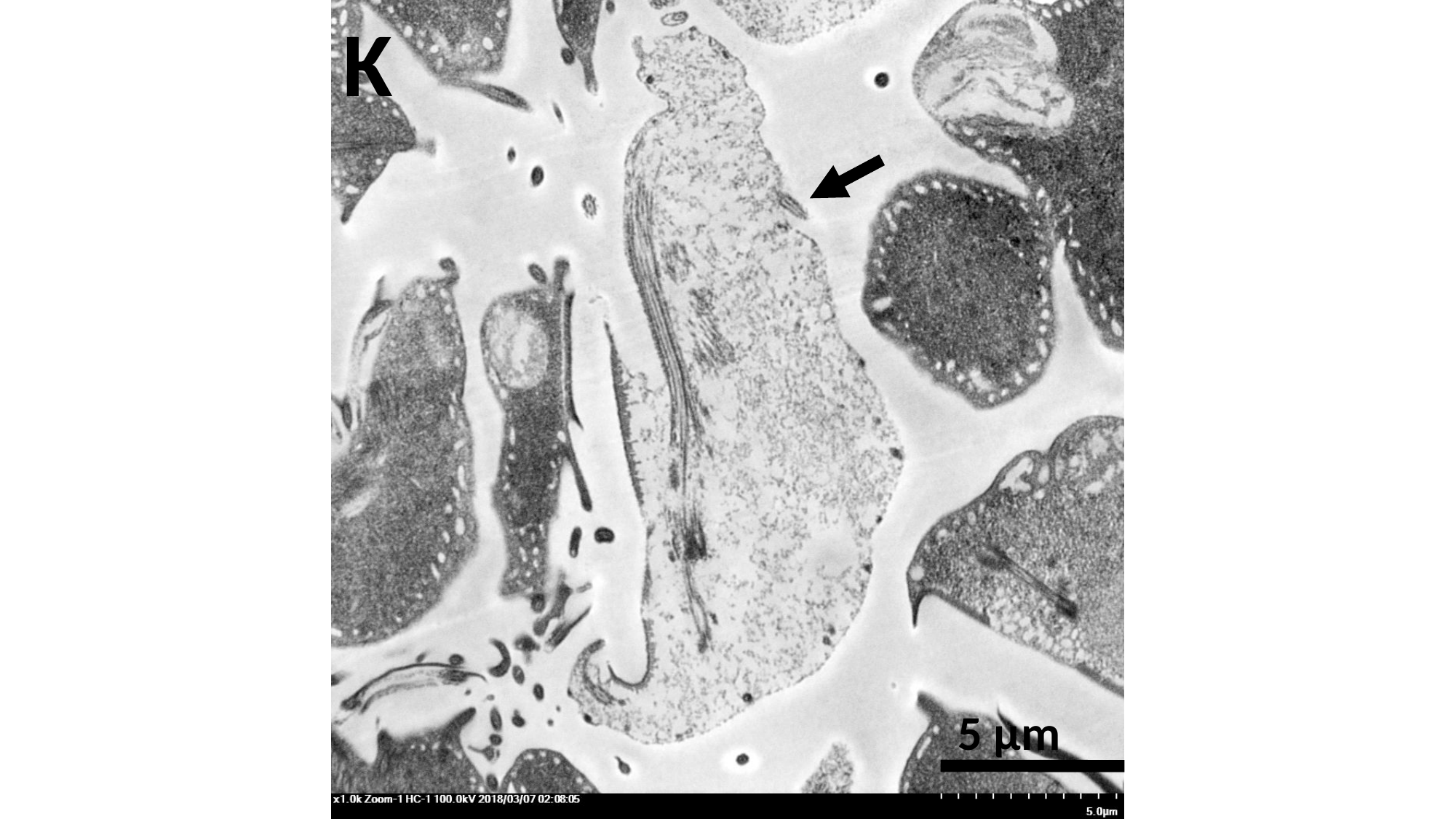

K
5 µm
